# Dietary Betaine Attenuates High-Carbohydrate-Diet-Induced Oxidative Stress, Endoplasmic Reticulum Stress, and Apoptosis in Mandarin Fish (*Siniperca chuatsi*)

**DOI:** 10.3390/antiox12101860

**Published:** 2023-10-13

**Authors:** Hongyan Li, Yanzhi Zeng, Xinyu Zheng, Guangjun Wang, Jingjing Tian, Wangbao Gong, Yun Xia, Kai Zhang, Zhifei Li, Wenping Xie, Jun Xie, Ermeng Yu

**Affiliations:** 1 Key Laboratory of Tropical & Subtropical Fishery Resource Application & Cultivation, Pearl River Fisheries Research Institute, Chinese Academy of Fishery Sciences, Guangzhou 510380, China; lihongyan@prfri.ac.cn (H.L.); zengyanzhi@prfri.ac.cn (Y.Z.); zhengxy@bces.ac.cn (X.Z.); gjwang@prfri.ac.cn (G.W.); tianjj@prfri.ac.cn (J.T.); gongwb@prfri.ac.cn (W.G.); xy@prfri.ac.cn (Y.X.); zk@prfri.ac.cn (K.Z.); lzf@prfri.ac.cn (Z.L.); xiewp@prfri.ac.cn (W.X.); xj@prfri.ac.cn (J.X.); 2 National Demonstration Center for Experimental Fisheries Science Education, Shanghai Ocean University, Shanghai 201306, China

**Keywords:** mandarin fish, high carbohydrate diet, betaine, oxidative stress, ER stress

## Abstract

To investigate the impact of betaine on high-carbohydrate-diet-induced oxidative stress and endoplasmic reticulum (ER) stress, mandarin fish (*Siniperca chuatsi*) (23.73 ± 0.05 g) were fed with control (NC), betaine (BET), high carbohydrate (HC), and high carbohydrate + betaine (HC + BET) diets for 8 weeks. The results showed that betaine significantly promoted the growth of mandarin fish irrespective of the dietary carbohydrate levels. The HC diet induced oxidative stress, as evidenced by significantly elevated MDA levels. The HC diet significantly stimulated the mRNA levels of genes involved in ER stress (*ire1*, *perk*, *atf6*, *xbp1*, *eif2α*, *atf4*, *chop*), autophagy (*ulk1*, *becn1*, *lc3b*), and apoptosis (*bax*). However, betaine mitigated HC-diet-induced oxidative stress by modulating antioxidant enzymes and alleviated ER stress by regulating the mRNA of genes in the PERK-eIF2a-ATF4 pathway. Additionally, betaine significantly reduced the mRNA levels of *becn1* and *bax*, along with the apoptosis rate, indicating a mitigating effect on autophagy and apoptosis. Overall, dietary betaine improved growth, attenuated HC-diet-induced oxidative stress and ER stress, and ultimately alleviated apoptosis in mandarin fish. These findings provide evidence for the use of betaine in aquafeeds to counter disruptive effects due to diets containing high carbohydrate levels.

## 1. Introduction

As an important non-protein energy source, the use of carbohydrates in feed can not only spare protein but also reduce the dependence on marine-derived ingredients [1]. Consequently, carbohydrates are commonly incorporated into aquafeeds due to their affordability and easy accessibility [1,2]. However, fish exhibit a poor ability to utilize dietary carbohydrates [3]. High carbohydrate (HC) diets have been found to negatively affect growth performance, metabolic activities, and immune responses in fish [4,5,6]. Notably, carnivorous fish are more vulnerable to dietary carbohydrates compared with omnivorous and herbivorous fish, as characterized by a persistent postprandial hyperglycemia after consuming a diet rich in carbohydrates [7,8,9]. Therefore, understanding the mechanisms underlying HC-diet-induced metabolic disorders in carnivorous fish is of great importance in the study of carbohydrate utilization in fish nutritional research.

Accumulating evidence has explored the potential mechanisms of HC-diet-induced adverse effects, one of which was emphasized in the oxidative stress and endoplasmic reticulum (ER) stress pathways [6]. The ER, the major site of protein synthesis, folding, and modification, is essential for cell function and survival [10]. Under conditions of internal or external stimuli, the aggregation of unfolded proteins disrupts cell homeostasis and leads to the occurrence of ER stress [10,11]. Subsequently, ER stress leads to the activation of the unfolded protein response (UPR) as a self-protective mechanism to restore ER homeostasis through three ER transmembrane receptors, including eukaryotic translation initiation factor 2-alpha kinase 3 (PERK), activating transcription factor 6 (ATF6), and inositol-requiring enzyme 1 (IRE1) [12,13]. However, prolonged or excessive ER stress could trigger the autophagy to engulf the damaged ER with the autophagic vesicles or even result in cell death through apoptosis [13]. Therefore, the ER stress, autophagy, and apoptosis pathways are orchestrated to maintain cellular homeostasis in organisms in the face of oxidative stress. Moreover, it has been reported that an HC diet induces oxidative stress and ER stress-induced autophagy and apoptosis in several fish species, such as largemouth bass (*Micropterus salmoides*) [14], golden pompano (*Trachinotus ovatus*) [15], and blunt snout bream (*Megalobrama amblycephala*) [16].

Currently, the implementation of feed additives with antioxidant properties is deemed an effective strategy to alleviate HC-diet-induced oxidative stress in fish. For example, studies have shown that dietary taurine and mulberry leaf flavonoids can improve growth performance and alleviate HC-diet-induced oxidative stress and ER stress in hybrid grouper (♀ *Epinephelus fuscoguttatus* × ♂ E. *lanceolatus*), turbot (*Scophthalmus maximus* L.), and eel (*Monopterus albus*) [17,18,19]. Betaine, also known as trimethylglycine, is a naturally occurring substance that was originally discovered as a by-product of sugar beet (*Beta vulgaris*) [20]. Betaine has many beneficial properties for growth performance, meat quality, and antioxidant and immune functions in the livestock or poultry industry [21]. In fish, betaine has traditionally been used as a feed attractant to promote feed intake by stimulating the olfactory bulb [22]. However, recent evidence suggests that betaine may also improve antioxidant capacity by regulating the activities of antioxidant enzymes such as superoxide dismutase (SOD) and catalase (CAT) [23,24]. Additionally, betaine has been found to reduce endoplasmic reticulum stress and apoptosis [25]. Nevertheless, it is unclear whether dietary betaine supplementation could alleviate the oxidative stress, ER stress, and apoptosis induced by a high carbohydrate diet in fish.

The mandarin fish (*Siniperca chuatsi*) is a high-economic freshwater species with unique feeding habitats. To date, mandarin fish have been reported to be able to consume artificially formulated diets well after domestication [26,27]. As a typical carnivorous species, mandarin fish had a limited capacity to utilize dietary carbohydrates, making them a valuable model for investigating carbohydrate utilization in teleost fishes. Previous research has shown that mandarin fish exhibit anorexic behavior when exposed to a diet containing 8% carbohydrate for prolonged periods [28]. Additionally, mandarin fish displayed metabolic abnormalities, including hyperglycemia and excessive lipid accumulation, when subjected to a high carbohydrate diet [29]. However, the effects of HC diets on oxidative stress, ER stress, and apoptotic responses have rarely been investigated in mandarin fish, and whether betaine supplementation can ameliorate such negative effects is warranted. Therefore, the present study was conducted to investigate the effects of a high carbohydrate diet and the potential mitigating mechanisms of betaine supplementation on oxidative stress, ER stress, and associated autophagy and apoptosis in mandarin fish. This study will provide new insights into carbohydrate utilization in fish, establish a theoretical basis for the implementation of betaine in aquatic feeds, and ultimately contribute to the promotion of a healthy mandarin fish aquaculture industry.

## 2. Materials and Methods

### 2.1. Experimental Diets

Four experimental diets were formulated and coded as control (NC), betaine (1%, Solarbio) (BET), high carbohydrate (HC, 20%), and high carbohydrate (20%) + betaine (1%) (HC + BET) diet groups. All diets were isonitrogenous and isolipic with fishmeal and fish oil as the protein and lipid sources, respectively. The diets were formulated to meet the nutritional requirements of mandarin fish, and the carbohydrate content of the HC diet was set based on Zhang et al., [29]. Betaine (with a purity of ≥98.0%) was obtained from Beijing Solarbio Science & Technology Co., Ltd. (IB0150, Solarbio, Beijing, China). The formulation and proximate composition of the experimental diets are shown in Table 1. The ingredients were finely ground and thoroughly mixed before being processed into pellets using a pellet machine. All diets were air-dried and stored at −20 °C until use.

The proximate composition of the experimental diets was determined according to AOAC (2003) methods [30]. Briefly, crude protein content was determined using the Kjeldahl nitrogen assay after acid digestion. Crude lipid content was determined using a 4800 Kjeltec Analyzer Unit (FOSS Tecator, Haganas, Sweden). Moisture content was determined by drying the samples to constant weight at 105 °C in an oven. Ash content was determined after calcination in a muffle furnace at 550 °C to constant weight.

### 2.2. Fish and Feeding Trial

The experiment was conducted in an indoor rearing system at the Pearl River Fisheries Research Institute, Guangzhou, China. Juvenile mandarin fish (*Siniperca chuatsi*) that were well-domesticated (could successfully consume artificial diets) were reared on the control diet for two weeks prior to the formal trial to acclimate to the experimental conditions. All experimental fish were then fasted for 24 h, and 300 healthy mandarin fish (initial weight 23.73 ± 0.05 g) were randomly divided into four groups, with three tanks in triplicate and 25 fish in each tank. The experimental fish were then fed with the four groups of diets to apparent satiation twice a day (at 08:30 and 16:00) for 8 weeks. Water quality was monitored regularly throughout the eight-week rearing period. Temperature was maintained at 27.0–28.5 °C, the pH at 7.0–8.5, dissolved oxygen at 4.6–5.5 mg L^−1^, and total ammonia nitrogen below 0.1 mg L^−1^.

### 2.3. Sample Collection

At the end of the feeding trial, fish were anesthetized with MS-222 (60 mg/L; E10521, Sigma, St. Louis, USA), counted, and then batch-weighed to calculate the average of final body weight (FBW). The feeding rate (FR) and feed efficiency (FE) were calculated from IBW, FBW, and food intake using a specific formula (See footnote for Table 3). For sampling, three fish were selected from each tank to measure the length and weight of the fish in order to calculate the conditional factor (CF). The viscera and hepatopancreas were removed from these fish and weighted to calculate the viscerosomatic index (VSI) and the hepatosomatic index (HSI). Two fish were randomly selected from each tank and blood was drawn carefully from the tail vein using a sterile syringe washed with heparin sodium (0.02%, Aladdin, Shanghai, China). The supernatant was collected after centrifugation in a sterile centrifuge tube at 4000 rpm for 10 min. Livers were immediately dissected in an ice bath and stored at −80 °C for subsequent analysis.

### 2.4. Enzyme Activities

The enzyme activities of total antioxidant capacity (T-AOC, A015-2-1), malondialdehyde (MDA, A003-1-2), superoxide dismutase (SOD, A001-3-2), and catalase (CAT, A007-1-1) in the livers of mandarin fish were determined using commercial kits from the Nanjing Jiancheng Bioengineering Institute (Nanjing, China) according to the manufacturer’s instructions.

### 2.5. Histological Analysis

Terminal-deoxynucleotidyl transferase-mediated nick end labeling (TUNEL) assays were performed to observe apoptotic signals by detecting DNA fragmentation in the livers of mandarin fish. The assays were performed according to the methods described by Li et al. [11]. Images were observed in digital images from a Nikon Eclipse Ti-SR inverted microscope.

### 2.6. Quantitative Real-Time PCR

Total RNA was extracted from livers using Trizol reagent (15596026, Invitrogen, Carlsbad, CA, USA). The purity and integrity of the RNA was confirmed with agarose gel electrophoresis, and the concentration was determined using an Implen NanoPhotometer (Implen Inc., Munich, Germany). Subsequently, cDNA was synthesized using a Takara reverse transcription kit (2690A, Takara, Shiga, Japan) according to the manufacturer’s protocol. Quantitative real-time PCR (qRT-PCR) test was performed using a Light Cycler^®^ 96-Time PCR system (Roche, Switzerland). Reaction systems were set to a volume of 20 μL, consisting of 0.8 μL of each primer (10 μM), 6.4 μL of sterilized ddH_2_O, 2 μL of diluted first-strand cDNA product, and 10 μL 2 × SYBR Premix Ex Taq II (RR390A, TaKaRa, Shiga, Japan). Cycling procedures were as follows: 95 °C for 5 min, followed by 45 cycles of 95 °C for 15 s and 60 °C for 1 min. Negative controls were established by using the non-cDNA and DNase-treated non-reverse transcribed tissue RNA samples. Table 2 shows the primer sequences used in this experiment, and *β-actin* and *rpl13α* were employed as the housekeeping genes. Relative gene expression was calculated based upon Pfaffl’s mathematical model [31].

### 2.7. Statistical Analysis

Data analysis was performed using SPSS 22.0 software (SPSS Inc., Chicago, IL, USA). All data were expressed as mean ± standard error of the mean (SEM). One-way ANOVA analysis was performed, followed by Duncan’s multiple comparisons between groups (NC vs. BET, NC vs. HC, HC vs. HC + BET). A value of *p* < 0.05 indicates a statistically significant difference.

## 3. Results

### 3.1. Growth Performance, Feed Utilization, and Morphological Indices

After 8 weeks of the feeding experiment, the final body weight (FBW) and specific growth rate (SGR) were significantly increased in the BET group, as compared with the NC group (Table 3, *p* < 0.05). Mandarin fish fed with the HC diet had significantly higher SGRs and condition factors (CFs) than those in the NC group. In addition, dietary betaine increased the FBW and SGR of mandarin fish in the HC + BET group as compared with the HC group (*p* < 0.05). No significant differences in feed efficiency (FE), feeding rate (FR), and other morphological indices were observed between the groups (*p* > 0.05).

### 3.2. Hepatic Antioxidant Enzyme Activities

**Figure 1:**
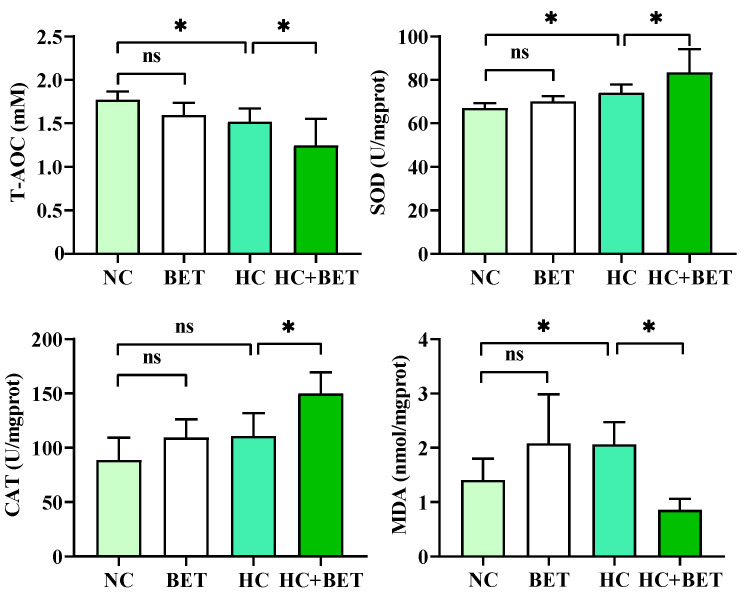
Effects of dietary betaine on antioxidant enzyme activities in the livers of mandarin fish fed the HC diet. Data are expressed as mean ± SEM (*n* = 6). * indicates significant difference between groups (*p* < 0.05). T-AOC: total antioxidant capacity; SOD: superoxide dismutase; CAT: catalase; MDA: malondialdehyde.

The activities of antioxidant enzymes in the livers of mandarin fish after the 8-week feeding trial are shown in Figure 1. Compared with the NC group, the HC diet induced significantly lower T-AOC levels but higher SOD and MDA levels in the livers of mandarin fish (*p* < 0.05). The use of betaine in the HC diet stimulated the CAT and SOD activities but reduced T-AOC and MDA levels compared with those in the HC group (*p* < 0.05).

### 3.3. Antioxidant-Related Gene Expression in the Liver

**Figure 2:**
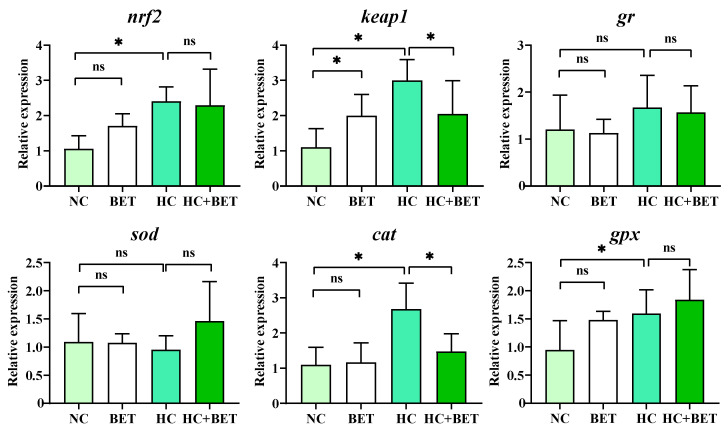
Effects of dietary betaine on hepatic antioxidant-related genes in mandarin fish fed the HC diet. Data are expressed as mean ± SEM (*n* = 6). * indicates significant difference between groups (*p* < 0.05). *nrf2*: nuclear factor erythroid 2-related factor; *keap1*: kelch-like ECH-associated protein 1; *gr*: glutathione reductase; *sod*: superoxide dismutase; *cat*: catalase; *gpx*: glutathione peroxidase.

The expression of the genes involved in antioxidant systems in the livers of mandarin fish is shown in Figure 2. Compared with the NC group, the mRNA levels of *nrf2*, *keap1*, *cat*, and *gpx* in the HC group and *keap1* in the BET group were significantly elevated (*p* < 0.05). In addition, the mRNA levels of *keap1* and *cat* were significantly decreased in the HC + BET group compared with those in the HC group (*p* < 0.05). There were no significant differences in the mRNA levels of *gr* and *sod* between the groups (*p* > 0.05).

### 3.4. ER-Stress-Related Gene Expression in the Liver

**Figure 3:**
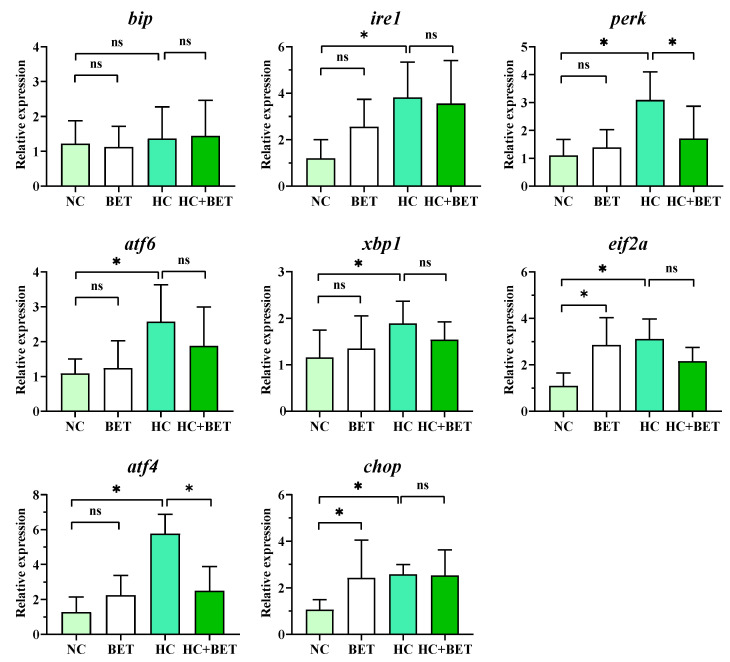
Effects of dietary betaine on hepatic genes involved in ER stress in mandarin fish fed the HC diet. Data are expressed as mean ± SEM (*n* = 6). * indicates significant difference between groups (*p* < 0.05). *bip*: GRP78 immunoglobulin heavy chain-binding protein; *ire1*: inositol-requiring protein-1; *perk*: eukaryotic translation initiation factor 2-alpha kinase 3; *atf6*: activating transcription factor 6; *xbp1*: x-box binding protein 1; *eif2a*: eukaryotic translation initiation factor 2a; *atf4*: activating transcription factor 4; *chop*: DNA damage inducible transcript 3.

The expression of genes involved in ER stress in the livers of mandarin fish is shown in Figure 3. Compared with the NC group, the HC diet upregulated the transcript levels of *ire1*, *perk*, *atf6*, *xbp1*, *eif2a*, *atf4*, and *chop*. Meanwhile, the mRNA levels of *eif2a* and *chop* were significantly increased in the BET group compared with the NC group (*p* < 0.05). Furthermore, the expression levels of *perk* and *atf4* were significantly down-regulated in the HC + BET group compared with those in the HC group (*p* < 0.05). No significant difference in the transcript level of *bip* was found between the groups (*p* > 0.05).

### 3.5. Autophagy-Related Gene Expression in the Liver

**Figure 4:**
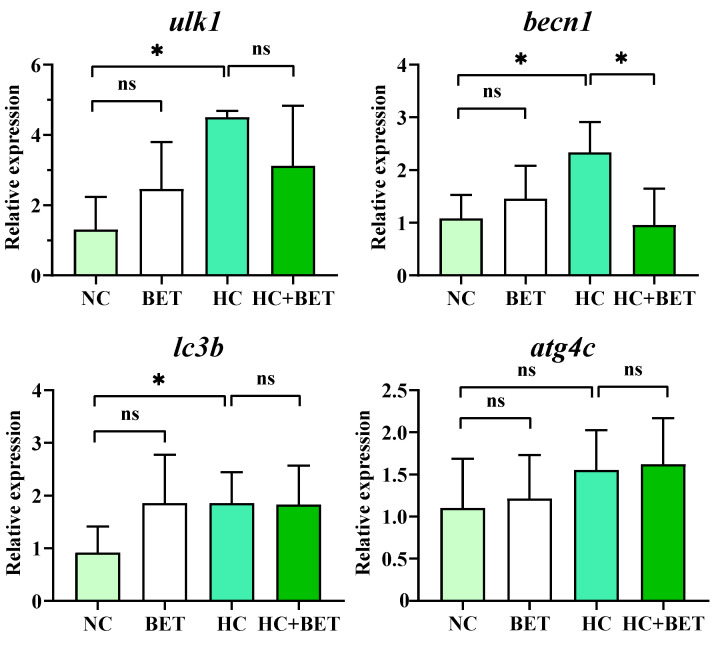
Effects of dietary betaine on hepatic genes involved in autophagy in the livers of mandarin fish fed the HC diet. Data are expressed as mean ± SEM (*n* = 6). * indicates significant difference between groups (*p* < 0.05). *ulk1*: Unc-51 like autophagy activating kinase 1; *becn1*: beclin 1; *lc3b*: microtubule-associated protein 1 light chain 3b; *atg4c*: autophagy related 4c cysteine peptidase.

The expression of genes involved in autophagy in the livers of mandarin fish is shown in Figure 4. Compared with the NC group, the mRNA levels of *ulk1*, *becn1*, and *lc3b* were significantly increased in the HC group (*p* < 0.05). Meanwhile, the transcript level of *becn1* was significantly decreased in the HC + BET group compared with that in the HC group (*p* < 0.05). The mRNA levels of *atg4c* were unchanged in all the groups (*p* > 0.05).

### 3.6. Apoptosis-Related Gene Expression and TUNEL Observations in the Liver

**Figure 5:**
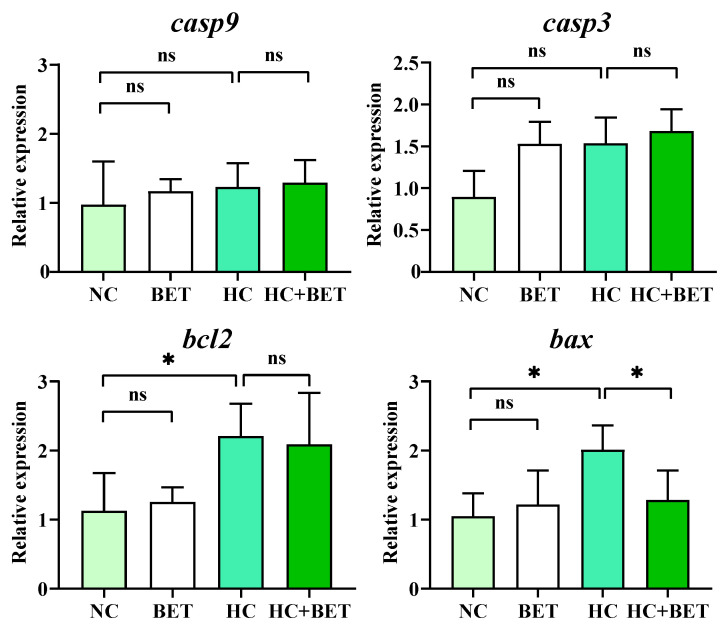
Effects of dietary betaine on hepatic genes involved in apoptosis in the livers of mandarin fish fed the HC diet. Data are expressed as mean ± SEM (*n* = 6). * indicates significant difference between groups (*p* < 0.05). *casp9*: caspase 9; *casp3*: caspase 3; *bcl2*: B-cell lymphoma-2; *bax*: Bcl2-associated X protein.

The expression of genes involved in apoptosis in the livers of mandarin fish is shown in Figure 5. Compared with the NC group, the mRNA levels of *bcl2* and *bax* were significantly increased in the HC group (*p* < 0.05). The transcript level of *bax* was significantly lower in the HC + BET group than in the HC group (*p* < 0.05). No significant differences were observed in the mRNA levels of *casp9* and *casp3* between all groups (*p* > 0.05).

**Figure 6:**
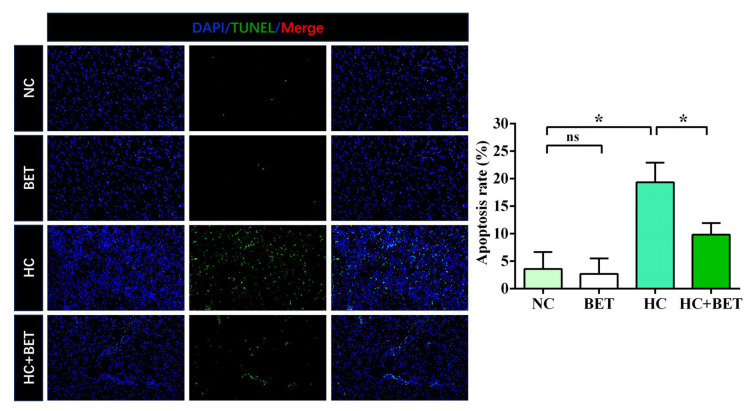
Representative DAPI and TUNEL staining of sections of mandarin fish liver. The microscope magnification was 200×. Positive apoptotic nuclei and normal nuclei are shown in green and blue, respectively. Apoptotic index: the number of apoptotic nuclei/the number of observed nuclei × 100%. Data are expressed as mean ± SEM (*n* = 6). * indicates significant difference between groups (*p* < 0.05).

The results of TUNEL and DAPI staining in the livers of mandarin fish are presented in Figure 6. The results showed that the apoptosis signals and the apoptosis rate were significantly higher in the HC group than in the NC group. Meanwhile, betaine supplementation significantly reduced the apoptosis rate in the HC + BET diet group compared with the HC group (*p* < 0.05).

## 4. Discussion

### 4.1. Betaine Promoted Growth of Mandarin Fish Regardless of Dietary Carbohydrate Levels

Dietary carbohydrates are becoming increasingly important in aquaculture due to the rapid growth of aquaculture production and the limited supply of protein sources. Aquafeeds with appropriate levels of carbohydrates can facilitate feed pelleting, reduce the catabolism of proteins and lipids for energy, and promote fish growth [7,32]. However, excessive dietary carbohydrates always have a negative effect on the growth of many fish species, including eel and largemouth bass [5,33]. On the contrary, some studies have also demonstrated that high carbohydrate diets have no effect on growth, such as in the Indian major carp (*Cirrhinus mrigala*) and rainbow trout (*Oncorhynchus mykiss*) [34,35]. In this study, the HC diet induced a significantly higher SGR than the NC diet, indicating that the HC diet promoted the growth performance of mandarin fish. This result was in line with a previous study that mandarin fish achieved maximum SGRs when fed a high carbohydrate diet [29]. The differential effects on growth between the NC and HC diets can be attributed to their varying energy levels resulting from different carbohydrate contents. Additionally, factors such as fish age, species, and environmental conditions are also likely contributors to these observed differences [36].

Despite the effects of the HC diet on growth, apparent impacts on growth performance were also found in mandarin fish fed a diet containing betaine. Betaine is known to be a dietary stimulant that has been proposed to improve growth in fish [20]. Previous studies have shown that dietary betaine supplementation improves growth performance in many aquatic animals, such as gibel carp (*Carassius auratus gibelio*), tilapia (*Oreochromis niloticus*), blunt snout bream, and *Macrobrachium rosenbergii* [21,23,37,38]. Similarly, dietary betaine supplementation significantly increased the FBW and SGR of mandarin fish in the BET and HC + BET groups compared with the NC and HC groups, respectively. This indicates that irrespective of the quantity of carbohydrates included in the diet, betaine supplementation has the potential to enhance body weight and facilitate growth in mandarin fish. Taken together, the results indicated that dietary betaine at a level of 1% can promote the growth performance of mandarin fish, irrespective of the dietary carbohydrate level.

### 4.2. Betaine Mitigated HC-Diet-Induced Oxidative Stress in Mandarin Fish

Reactive oxygen species (ROS) in organisms are normally maintained in a dynamic equilibrium with the contribution of the antioxidant scavenging systems, whereas nutritional imbalances could disrupt the homeostasis of the pro-oxidant–antioxidant system, leading to oxidative stress [19,39]. Fish have evolved antioxidant defense mechanisms to counteract stress, particularly through Nrf2-Keap1 signaling [11]. Nrf2-Keap1 signaling is an evolutionarily conserved intracellular defense mechanism to counteract oxidative stress by regulating the activities of antioxidant enzymes [40]. Among these enzymes, T-AOC represents the ability of all enzymatic and non-enzymatic antioxidants to scavenge free radicals to protect against oxidative damage. The antioxidant enzyme SOD converts free radicals to oxygen and hydrogen peroxide, which is then catalyzed by CAT to form water and oxygen [15]. In the present study, the mRNA levels of *nrf2/keap1* and its downstream genes of *cat* and *gpx* were significantly increased in mandarin fish fed the HC diet, which was inconsistent with findings in other species such as largemouth bass [14] and blunt snout bream [41]. However, divergence between the gene transcription and enzyme activity may occur due to the presence of post-translation modification. The HC diet significantly reduced T-AOC levels but increased MDA contents in the livers of mandarin fish compared with the NC diet. MDA is considered a vital biomarker of lipid peroxidation under oxidative stress, which confirmed the occurrence of oxidative stress in mandarin fish fed the HC diet. Taken together, the HC diet stimulated the antioxidant system to counteract the disruption of redox homeostasis to some extent but failed to maintain the balance and ultimately caused oxidative stress in mandarin fish. This result was consistent with previous studies in which a high carbohydrate diet reduced antioxidant capacity and induced oxidative stress in largemouth bass [42] and blunt snout bream [43]. In addition, dietary betaine has been reported to enhance antioxidant capacity in blunt snout bream under an unbalanced nutrient status [23]. Similarly, dietary betaine supplementation in the HC diet stimulated the activities of SOD and CAT and reduced the MDA levels in the livers of mandarin fish, suggesting an ameliorative effect of betaine in HC-diet-induced oxidative stress. Overall, the HC diet induced oxidative stress in the livers of mandarin fish, and the use of betaine in the diet attenuated oxidative stress by stimulating the antioxidant responses.

### 4.3. Betaine Alleviated HC-Diet-Induced Apoptosis by Attenuating ER Stress and Autophagy

Endoplasmic reticulum stress and oxidative stress are often simultaneous events, as the hydrogen peroxide generated by oxidative stress can transmit a stress signal to disrupt ER homeostasis, thereby triggering ER stress [11]. In mammals, carbohydrate overconsumption is a major dietary contributor to ER stress and lipid accumulation [44]. Despite the production of ROS, it has also been reported that excessive dietary carbohydrates caused the accumulation of unfolded proteins and induced ER stress in largemouth bass and turbot [14,18]. In the present study, the HC diet had significantly higher mRNA levels of *ire1*, *perk*, *atf6*, *xbp1*, *eif2a*, *atf4*, and *chop* in the livers of mandarin fish than in the NC group, indicating that the HC diet induced ER stress through the modulation of the PERK-eIF2a-ATF4 and IRE1-XBP1 pathways. Furthermore, autophagy is a highly conserved cytoprotective process that plays an indispensable role in determining the cell fate of an organism [45], while apoptosis is a highly controlled programmed cell death that occurs in multicellular organisms and can be triggered by a variety of extrinsic and intrinsic stress signals [46]. Prolonged ER stress links autophagy and apoptosis through the regulation of ATF4 and CHOP, which have been shown to modulate the transcription of ATG and the BCL2 protein family [47,48]. Ulk1, also known as ATG1, is the upstream gene of LC3b, both of which are key genes involved in the formation of phagocytes [45]. The HC diet increased the transcript levels of *ulk1*, *becn1*, and *lc3b* compared with the NC group, implying the activation of autophagic processes in the livers of mandarin fish. In addition, the pro-apoptotic indicator *bax* was significantly higher in the HC group of mandarin fish, which was in line with the results in largemouth bass [14] that the HC diet induced apoptosis in the liver. The significantly upregulated mRNA levels of apoptosis genes in the HC group were further confirmed by the significantly obvious apoptosis signals and apoptosis rate in the livers of mandarin fish using the TUNEL assay. As a whole, the HC diet induced ER stress and subsequently stimulated autophagic progression, leading to apoptosis in mandarin fish, as compared with the NC diet.

The use of dietary antioxidants has been widely used in aquafeed to prevent aquatic animals from suffering from environmental stress or nutritional imbalance [15,17,18]. Regarding the effects of dietary betaine use in diets, significantly lower mRNA levels of *eif2a* and *chop* in the BET group and *perk* and *atf4* in the HC + BET group were observed in the livers of mandarin fish, compared with the NC and HC groups, respectively. Therefore, betaine in the HC diet significantly alleviated ER stress by regulating the PERK-eIF2a-ATF4 pathway (a branch of the UPR pathway) in the livers of mandarin fish. Since ER stress is a potent trigger for autophagy [11], the suppression of ER stress also induced the down-regulation of autophagy, as indicated by the significantly lower level of *becn1* in the livers of mandarin fish fed the HC + BET diet. Moreover, CHOP is a non-ER-localized transcription factor of the ER stress response, which is stimulated by the activation of PERK-eIF2α-ATF4 and then activates apoptosis by upregulating the expression of pro-apoptotic genes such as BAX [46]. In the present study, the transcriptional levels of *bax* were significantly lower in the HC + BET group than in the HC group. In addition, betaine supplementation significantly reduced the apoptosis rate in the livers of mandarin fish compared with the HC diet, as shown by histological observation, indicating an alleviating effect on apoptosis in the livers of mandarin fish fed the HC diet with betaine. Therefore, the suppression of ER stress was positively correlated with the reduction in autophagy, which ultimately led to the alleviation of apoptosis. Taken together, dietary betaine supplementation reduced the HC-diet-induced apoptosis via attenuating the ER stress and autophagy in mandarin fish.

## 5. Conclusions

**Figure 7:**
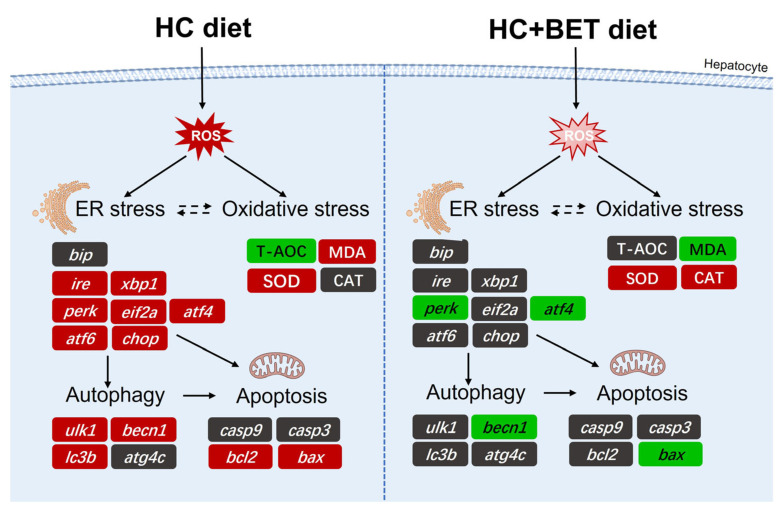
Schematic overview of the effects of dietary betaine on the HC-diet-induced oxidative stress, ER stress, and associated autophagy and apoptosis in the livers of mandarin fish. Red and green boxes represent up- and down-regulated genes/enzymes, respectively. The gray box indicates that no significant changes occurred.

Our findings demonstrated that dietary betaine at a level of 1% significantly improved the growth of mandarin fish, irrespective of the dietary carbohydrate level. The HC diet induced oxidative stress, ER stress, and its associated autophagy, ultimately leading to apoptosis in the livers of mandarin fish. In addition, dietary betaine attenuated HC-diet-induced oxidative stress by activating antioxidant enzymes such as CAT and SOD in the livers of mandarin fish. The use of betaine in the HC diet significantly alleviated ER stress by regulating the PERK-eIF2a-ATF4 pathway, mitigated the autophagic process via *becn1*, and attenuated apoptosis in the livers of mandarin fish (Figure 7). Collectively, our findings provide new insights into the potential application of betaine in aquafeeds to alleviate the disruptive effects induced by an HC diet in teleost fish.

## Figures and Tables

**Table 1:** Formulation and proximate composition of the experimental diets.

Ingredient (%)	Diets			
	NC	BET	HC	HC + BET
Fish meal	70	70	70	70
Corn starch	0	0	20	19
Fish oil	3	3	3	3
Vitamin mix ^1^	2	2	2	2
Mineral mix ^2^	2	2	2	2
Microcrystalline cellulose	20	19	0	0
Carboxymethylcellulose sodium	3	3	3	3
Betaine ^3^	0	1	0	1
Proximate composition				
Crude protein (%)	45.08	45.98	45.48	45.69
Crude lipid (%)	8.02	7.84	8.10	8.05
Moisture (%)	6.30	6.88	5.30	8.29
Ash (%)	16.11	15.83	16.09	15.59
Energy (kJ/g)	15.66	15.60	18.96	18.78

^1^ Vitamin mix: from Guangdong Nutriera Group, Guangzhou, China. Vitamin A 10; vitamin B_1_ 6; vitamin B_2_ 5; vitamin B_6_ 7.5; vitamin B_12_ (1%) 4; niacinamide 50; ascorbyl calcium phosphate (35%) 500; calcium pantothenate 20; biotin (2%) 2.5; folic acid 5; vitamin E (50%) 200; vitamin K3 10; vitamin D3 5; inositol 100; corn protein powder 75. ^2^ Mineral mix: from Guangdong Nutriera Group, Guangzhou, China. CuSO_4_·5H_2_O 10; FeSO_4_·H_2_O 300; ZnSO_4_·H_2_O 200; MnSO_4_·H_2_O 100; KIO_3_ (10%) 80; Na_2_SeO_3_ (10% Se) 67; CoCl_2_·6H_2_O (10% Co) 5; NaCl 100; zeolite 638. ^3^ Betaine: Beijing Solarbio Science & Technology Co., Ltd. (IB0150, Solarbio, Beijing, China).

**Table 2:** Sequences of primers used for qRT-PCR analysis.

Genes	Sequence (5′-3′)	Amplicon Size (bp)	Amplification Efficiency	Accession No.
Beta-actin (*β-actin*)	F: TGCGTGACATCAAGGAGAAGC	176	1.92	XM_044169301.1
	R: GAGGAAGGAAGGCTGGAAGAG			
Ribosomal protein L13a (*rpl13a*)	F: CACCCTATGACAAGAGGAAGC	100	2.01	MK770673
	R: TGTGCCAGACGCCCAAG			
Nuclear factor erythroid 2-related factor (*nrf2*)	F: ACGAAAGCGAAAGCTCCTCA	90	1.89	MT270449.1
	R: GCTCTCTTCCAGAATGGCGT			
Kelch-like ECH-associated protein 1 (*keap1*)	F: GTGGCAACCCAGGAGGAG	187	1.82	XM_044189604.1
	R: GGGAATGGCAACGGACA			
Glutathione reductase (*gr*)	F: CAGGCATCCTTTCCACCC	178	2.11	XM_044204922.1
	R: TCCAGTCCTCTGTCCGTTTTA			
Superoxide dismutase (*sod*)	F: CACGCTCCCTGACCTGACA	176	1.83	XM_044168059.1
	R: GGAGGGCAACCTGTGCTG			
Catalase (*cat*)	F: GCGTTTGGCTACTTTGAGGT	108	1.82	XM_044194118.1
	R: CACAGTGGAGAAGCGGACA			
Glutathione peroxidase (*gpx*)	F: GCCCATCCCCTGTTTGTG	185	1.92	XM_044172415.1
	R: AACTTCCTGCTGTAACGCTTG			
GRP78 immunoglobulin heavy chain-binding protein (*bip*)	F: GGCCACTAAGGATGCTGGAA	136	1.84	XM_044173053.1
	R: ACCACCCAAATCGAACACGA			
Inositol-requiring protein-1 (*ire1*)	F: CATACAGGTCAGTTTCTGCTACAC	106	1.81	XM_044184760.1
	R: AAATCAACATCCCTGCCACCT			
Eukaryotic translation initiation factor 2-alpha kinase 3 (*perk*)	F: TGCTGGAGTCATCCTACCGA	113	1.83	XM_044166231.1
	R: CGCAGAGCAGATGTACCGAA			
Activating transcription factor 6 (*atf6*)	F: AGATGAGTTGCTTGAGGCCC	150	1.86	XM_044198830.1
	R: GCAGGTGACAGAGAGTCCAC			
X-box-binding protein 1 (*xbp1*)	F: AAACAGGGTGCTTCGGGAAA	125	1.9	XM_044196969.1
	R: CATTCCCGGTGGACAACAGA			
Eukaryotic translation initiation factor 2A (*eif2a*)	F: CTGTGCACACCCCCTTATGT	241	1.79	XM_044178962.1
	R: CGTGGATGGACGGGTAATGT			
Activating transcription factor 4 (*atf4*)	F: TGCACTGGCTATTTCTGGCAA	131	1.92	XM_044187002.1
	R: ATTTGGTCATGCTTTGGCCG			
DNA-damage-inducible transcript 3 (*chop*)	F: AACGGTGCCTTGTCCACTTT	122	1.77	XM_044216843.1
	R: TCCGTCAGCTCCTGTACCTT			
Caspase 9 (*casp9*)	F: ACACAGGCTTTGAGGTGTCC	157	1.81	XM_044210980.1
	R: AGAATGTCACATGGGGTGGG			
Caspase 3 (*casp3*)	F: ACAGGTGCTACGCCTCATTC	172	1.82	GU178032
	R: CCTCTGCAAGCCTGGATGAA			
B-cell lymphoma-2 (*bcl2*)	F: CCAGAAAACATTCCACCAAAG	148	1.8	XM_044197230.1
	R: GGGAGATGAGTAAGGAAGGGA			
Bcl2-associated X protein (*bax*)	F: TCCTACTTTGGCACACCCAC	108	2.07	XM_044180218.1
	R: TGTCTGCTCTTCACGAACCC			
Unc-51 like kinase 1 (*ulk1*)	F: GTGCCTGCCCAGTTTCCC	268	1.76	XM_044195569.1
	R: GCAGGTTCTGTTCCATACGCT			
Beclin 1 (*becn1*)	F: AGGAGGTGAAGAGCGATAAGG	123	2.06	XM_044167873.1
	R: CCAGGCGACGGTTGTGA			
Microtubule-associated protein 1 light chain 3b (*lc3b*)	F: AGAGCAGCACCCCAGCAA	182	1.9	XM_044194970.1
	R: CGTTGACCAGCAGGAAGAAA			
Autophagy related 4c cysteine peptidase (*atg4c*)	F: TCAGCACCAGCGATTTCCC	181	2.15	XM_044200084.1
	R: GCGGGGTATTTCTCCTTCG			

**Table 3:** Effects of dietary betaine on growth performance, feed utilization, and morphological indices of mandarin fish fed the HC diet.

Parameter	Diets				NC vs. BET	NC vs. HC	HC vs. HC + BET
	NC	BET	HC	HC + BET			
IBW ^1^ (g)	23.58 ± 0.02	23.94 ± 0.14	23.75 ± 0.04	23.68 ± 0.10	ns	ns	ns
FBW ^2^ (g)	64.93 ± 4.71	87.67 ± 0.70	80.71 ± 3.12	100.43 ± 7.48	*	ns	*
SGR ^3^ (%/d)	1.81 ± 0.13	2.32 ± 0.02	2.18 ± 0.07	2.57 ± 0.13	*	*	*
FE ^4^ (%)	40.66 ± 3.37	48.32 ± 1.54	53.62 ± 3.89	68.33 ± 7.03	ns	ns	ns
FR ^5^ (%BW/d)	3.02 ± 0.28	2.80 ± 0.08	2.89 ± 0.29	2.61 ± 0.09	ns	ns	ns
CF ^6^ (g/cm^3^)	2.33 ± 0.13	2.52 ± 0.02	2.67 ± 0.07	2.64 ± 0.14	ns	*	ns
HIS ^7^ (%)	1.18 ± 0.06	1.30 ± 0.04	1.27 ± 0.02	1.33 ± 0.13	ns	ns	ns
VSI ^8^ (%)	8.49 ± 0.16	7.89 ± 0.46	8.36 ± 0.75	8.62 ± 0.62	ns	ns	ns

Data are expressed as mean ± SEM of three replicates (*n* = 3). Significant differences between groups are illustrated by * in the columns (*p* < 0.05). ^1^ IBW: initial body weight; ^2^ FBW: final body weight; ^3^ Specific growth rate (SGR, %/d) = [ln (final weight) − ln (initial weight)]/days × 100; ^4^ Feed efficiency (FE, %) = wet weight gain/dry feed intake; ^5^ Feeding rate (FR, %BW/d) = 100 × dry feed intake/(days × (FBW + IBW)/2); ^6^ Condition factor (CF, g/cm^3^) = body weight (g) × 100/body length (cm^3^); ^7^ Hepatosomatic index (HSI, %) = 100 × hepatopancreas weight (g)/body weight (g); ^8^ Viscerosomatic index (VSI, %) = 100 × visceral weight (g)/body weight (g).

## Data Availability

Data are contained within the article.
